# Analysis of Microbiota Structure and Potential Functions Influencing Spoilage of Fresh Beef Meat

**DOI:** 10.3389/fmicb.2020.01657

**Published:** 2020-07-22

**Authors:** Bo Kyoung Hwang, HyeLim Choi, Sang Ho Choi, Bong-Soo Kim

**Affiliations:** ^1^Department of Agricultural Biotechnology, Center of Food Safety and Toxicology, Seoul National University, Seoul, South Korea; ^2^Department of Life Science, Multidisciplinary Genome Institute, Hallym University, Chuncheon, South Korea

**Keywords:** beef microbiota, spoilage, potential functions, fermentation, core genera

## Abstract

Beef is one of the most consumed food worldwide, and it is prone to spoilage by bacteria. This risk could be caused by resident microbiota and their alterations in fresh beef meat during processing. However, scarce information is available regarding potential spoilage factors due to resident microbiota in fresh beef meat. In this study, we analyzed the microbiota composition and their predicted functions on fresh beef meat. A total of 120 beef meat samples (60 fresh ground and 60 non-ground beef samples) were collected from three different sites in South Korea on different months, and the microbiota were analyzed by the MiSeq system. Our results showed that although the microbiota in beef meat were varied among sampling site and months, the dominant phyla were the same with shared core bacteria. Notably, psychrotrophic genera, related to spoilage, were detected in all samples, and their prevalence increased significantly in July. These genera could inhibit the growth of other microbes with using glucose by fermentation. The results of this study extend our understanding of initial microbiota in fresh beef meat and potential functions influencing spoilage and can be useful to develop the preventive measures to reduce the spoilage of beef meat products.

## Introduction

Beef is one of the most commonly consumed meats worldwide, including Korea (Cho et al., 2010), however, beef products are highly perishable (Doulgeraki et al., 2012). Microorganisms in beef can cause the spoilage of products and food poisoning. Since beef meat is nutrient rich and has high water content, microorganisms from the processing environments can easily colonize beef meat (De Filippis et al., 2013). Even during storage in refrigeration temperatures, psychrotrophic bacteria such as lactic acid bacteria and *Pseudomonas* spp. can grow on beef meat, thereby increasing the risk of meat spoilage (Doulgeraki et al., 2012). In addition, outbreaks due to contamination of beef meat with *Escherichia coli* O157 and *Salmonella* spp. have continuously occurred despite the maintenance of high hygiene levels (Kivi et al., 2007; Friesema et al., 2012; Heiman et al., 2015). Several studies have analyzed spoilage bacteria and pathogens by culture-based methods to find ways to reduce spoilage and foodborne illness (Ercolini et al., 2006; Black et al., 2010; Limbo et al., 2010). Recent studies using high-throughput sequencing methods have also reported the presence of meat spoilage-related microorganisms and pathogens during processing steps or under different storage conditions (De Filippis et al., 2013; Hultman et al., 2015; Stoops et al., 2015; Yang et al., 2016). However, these studies focused on specific bacteria and provided limited information on the overall microbial composition of beef meat. Therefore, analyzing the whole microbiota associated with beef is essential to understanding the spoilage risk of fresh beef meat.

Furthermore, understanding the potential function of microbes in fresh beef meat before further processing is also important, since it is the initial status of microbiota and can influence the alteration of beef microbiota during further processes. Microorganisms generally interact with each other to maintain their functions (Freilich et al., 2010; Faust and Raes, 2012; Zheng et al., 2015). These interactions between microbes could be related to the spoilage in food products. Previous studies have also reported that two or more microorganisms contributed to spoilage simultaneously by interacting with each other (Borch et al., 1996; Jørgensen et al., 2000).

This study aimed to analyze the microbiota composition in fresh beef meat and their potential functions influencing the spoilage of meat and the alteration of microbiota during further processing. We compared the microbiota of fresh beef meat (ground and non-ground) collected from different sites in different seasons in South Korea by using the Illumina MiSeq sequencing. The effects of the environmental variables on bacterial distributions in beef meat were analyzed, and the spoilage risk was predicted using the information gathered from the results. The outcomes of the present study provide insights into initial microbiota in fresh beef meat and extended our understanding of spoilage by the microbiota in beef meat.

## Materials and Methods

### Sample Collection

A total of 120 beef samples (60 non-ground and 60 ground samples) were collected from the Livestock Processing Center (LPC; the government local livestock joint market) from three different sites (Supplementary Figure S1). These sites were the areas with maximum beef production in Korea based on the annual report of livestock production and marketing channel^1^. Cattle from different farms were gathered at the LPC of each site and processed, including slaughter. The beef meat was transferred to market or company for further processing. Therefore, the microbiota in beef meat from the LPC is an initial status of microbiota in fresh beef meat. To determine the influence of seasonal differences on beef microbiota, we collected the samples in January and July 2018. Four kilograms of the beef sample (10 non-ground and 10 ground samples at each site) were collected and transported in an ice box to the laboratory. Samples were stored at −80°C until further experiments.

### Metagenomic DNA Extraction

Non-ground beef was cut into 5 g cubes, and 5 cubes were selected randomly. Ground beef was homogenized, and 25 g of sample was randomly selected. The samples were diluted in 225 mL of buffered peptone water (10 g peptone, 5 g sodium chloride, 3.5 g disodium phosphate, and 1.5 g potassium dihydrogen phosphate; at pH 7.2). Bacterial cells were detached from beef using a spindle (microorganism homogenizer, Korea patent registration 10-2010-0034930) and stored at −80°C. Metagenomic DNA was extracted from each sample using the phenol DNA extraction method previously described (Lee et al., 2016). Extracted genomic DNA was purified with the PowerClean DNA Clean-up kit (MO Bio Laboratories, Carlsbad, CA, United States) and confirmed through 1% agarose gel electrophoresis.

### Qunatitative Real-Time Polymerase Chain Reaction

The bacterial amounts in each sample was estimated by quantitative real-time PCR of 16S rRNA genes as previously described (Lee et al., 2017; Kim et al., 2019). The amplification was performed using primers 340F (5′-TCC TACGGGAGGCAGCAG-3′) and 518R (5′-ATTACCGCG GCTGCTGG-3′) on a Thermal Cycler Dice Real Time System III (Takara Bio, Otsu, Japan). Triplicate reactions of each sample were conducted in a final volume of 25 μL containing 12.5 μL of 2 × SYBR Green PCR master mix (Bioneer, Korea), 2 μM of each primer, and 1 μL of a DNA template (10-fold dilution series of sample DNA) or distilled water (negative control) under the following conditions: 94°C for 5 min, followed by 40 cycles of denaturation at 94°C for 30 s, annealing at 55°C for 30 s, extension at 72°C for 30 s, and final extension at 72°C for 10 min. Standard curves were generated from parallel PCRs of serial log-concentrations (1 × 10^2^–1 × 10^8^) of 16S rRNA gene copy numbers of the *E. coli* K12 w3110 strain. Regression coefficients (*r*^2^) for all standard curves were ≥0.99. Differences between samples were determined with the Mann–Whitney *U*-test using R software ver.3.2.0. Values of *p* < 0.05 were considered statistically significant.

### MiSeq Sequencing

The extracted DNA was amplified using primers (targeting V1-V3 region of the 16S rRNA gene) with adapters (forward: 5′-adapter [TCGTCGGCAGCGTCAGATGTGT ATAAGAGACAG]-GAGTTTGATCMTGGCTCAG-3′; reverse: 5′-adapter [GTCTCGTGGGCTCGGAGATGTGTATAAGAG ACAG]-ATTACCGCGGCTGCTGG-3′). PCR amplification followed preparation of a 16S metagenomics sequencing library for the MiSeq system (Illumina, Inc., San Diego, CA, United States) was performed as described previously (Lee et al., 2017; Kim et al., 2019). The library was quantified using a PCR Thermal Cycler Dice Real-Time System III (Takara Bio.) with the GenNext NGS Library Quantification Kit (Toyobo, Osaka, Japan). Equimolar concentrations of each library from the different samples were pooled and sequenced using an Illumina MiSeq system (300 bp-paired ends), following the manufacturer’s instructions.

### Sequencing Data Analysis

The obtained sequences were analyzed using CLC genomic workbench (ver.11.0.1) with the Microbial Genomics Module (Qiagen) as previously described (Lee et al., 2017; Kim et al., 2019). Paired sequences were merged, and low-quality sequences (<430 bp of merged reads or quality score <30) and chimeric reads were removed using the USEARCH pipeline v.10.0.240^2^. Primer sequences were removed from the merged sequences, and sequences were clustered into operational taxonomic units (OTUs) by 97% sequence identity with the EzTaxon-e database (Yoon et al., 2017). The representative sequences in each OTU cluster were identified, their taxonomic position based on the EzTaxon-e database. To compare diversity indices, the numbers of reads in each sample were normalized by random subsampling and indices were calculated using MOTHUR (Schloss et al., 2009). Differences between samples were evaluated with the Mann–Whitney *U*-test and Kruskal–Wallis test in the R software. Results with *p* < 0.05 were considered statistically significant.

Canonical correspondence analysis (CCA) was performed to analyze the factors influencing the composition of microbiota using the Bray-Curtis distance matrix in the R software, while the significance was evaluated with the permutation test. To find the core genus in beef meat, the relative abundance of genera in each sample was used with the Venn package in R. The relative abundance of genera among samples was compared by the heat map using the pheatmap package in R. For this analysis, the genus with over 1% relative abundance in each sample was selected. Benjamini-Hochberg FDR was used to correct for multiple tests. Result with FDR < 0.01 were considered statistically significant.

### Prediction of Microbiota Function

The potential function of each group was predicted by the phylogenic investigation of communities by reconstruction of unobserved states 2(PICRUSt2) (Douglas et al., 2019). The abundance of the predicted function was normalized concerning 16S rRNA gene copy numbers, and MetaCyc pathways were used for analyzing predicted functions of microbiota. The statistical differences between groups were determined using the two-sided Welch’s test, and confidence intervals were calculated using the Welch’s inverted test in the Statistical Analysis of Metagenomic Profiles (STAMP) software (Parks and Beiko, 2010). Benjamini-Hochberg FDR was used to correct for multiple tests. Only significant results with *q*-value (corrected *p* < 0.01) were used.

## Results and Discussion

### Comparison of Diversity Indices and Bacterial Compositions Among Samples

**FIGURE 1:**
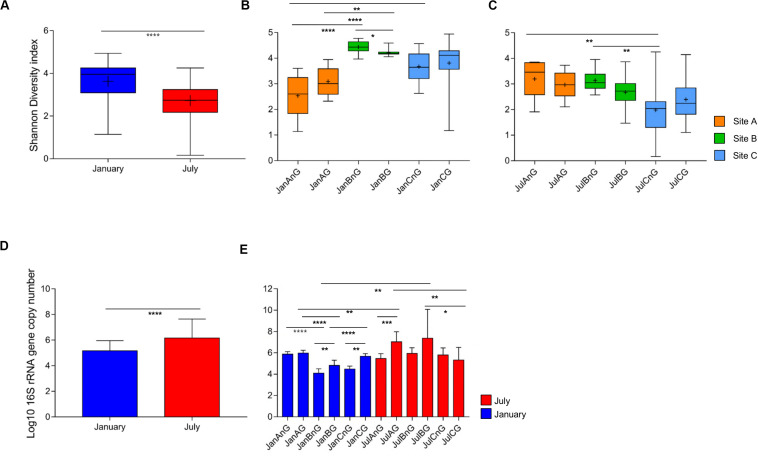
Comparison of bacterial diversity and abundance in beef meat samples obtained from different sites and times. **(A)** Comparison of bacterial diversity between samples obtained in January and July. **(B)** Comparison of diversity indices of samples obtained in January and **(C)** July. **(D)** The average bacterial abundance was compared between samples obtained in January and July. **(E)** The bacterial abundance in each group was compared. The sample name indicates the sampling month, site, and processing types (for example, JanAnG; sample collected in January from site A and non-ground beef). Error bars indicate the standard deviation. *p < 0.05, **p < 0.01, ***p < 0.001, ****p < 0.0001.

A total of 7,551,419 reads (average of 41,475 reads for January samples and 84,382 reads for July samples) were analyzed after the trimming process for the 120 beef samples (Supplementary Table S1). The numbers of observed OTUs were higher in the samples collected in July (average 61,041 ± 7,843) than in January (24,559 ± 1,922; *p* < 0.0001). The number of observed OTUs was highest in the ground beef from sampling site C in July (JulCG, 81,176 ± 39,019) and lowest in non-ground beef from site A in January (JanAnG, 11,415 ± 3,005). The Shannon diversity indices were compared between samples collected in January and July, as well as between samples from different sites at the same time. The average diversity of the microbiota was higher in samples collected in January (3.63 ± 0.12) than in July (2.73 ± 0.11) (*p* < 0.0001; Figure 1A). For January samples, the highest diversity was detected in non-ground beef from site B (4.44 ± 0.08), and the lowest diversity was detected in non-ground beef from site A (2.54 ± 0.25; *p* < 0.0001) (Figure 1B). For July, non-ground beef from site A had the highest diversity (3.20 ± 0.22), while non-ground beef from site C had the lowest diversity (1.99 ± 0.34; *p* < 0.01) (Figure 1C).

The relative bacterial abundance was determined and compared among samples using quantitative real-time PCR. The abundance of bacteria in beef samples was higher in July (average 1.45 × 10^6^ copies/g) than in January (average 1.44 × 10^5^ copies/g; *p* < 0.0001) (Figure 1D). The highest bacterial abundance was detected in ground beef from site B in July (average 2.39 × 10^7^ copies/g), while the lowest bacterial abundance was detected in non-ground beef from site B in January (average 1.23 × 10^4^ copies/g) (Figure 1E). These results indicate that the decreased diversity of July samples with higher bacteria abundance could be because of the dominance of some bacteria in the microbiota.

**FIGURE 2:**
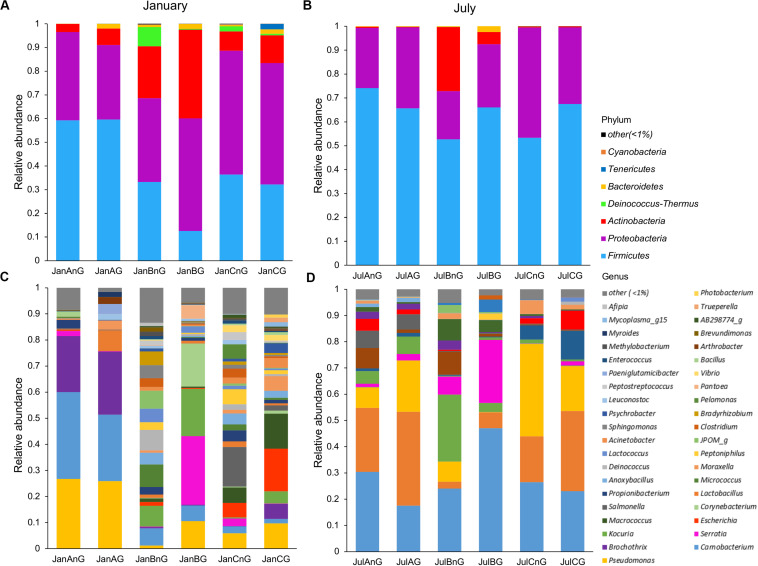
The composition of microbiota was compared among samples at the phylum and genus levels. Comparison of phylum composition in beef meat collected in **(A)** January and **(B)** July. Comparison of genus composition collected in **(C)** January and **(D)** July. Taxa with relative abundance <1% in each sample were combined with the “other” group.

The composition of microbiota in beef samples was compared at phylum and genus levels (Figure 2). *Firmicutes* (51.03%) and *Proteobacteria* (36.58%) were the dominant phyla in all beef samples. The proportions of *Firmicutes* were higher in July (average 63.20%) than in January (average 38.86%) (*p* < 0.05). Between the sites, site B revealed higher relative abundance of *Actinobacteria* both in January (average 14.93%) and July (average 29.67%) samples. However, the identified genera were more diverse in January than in July samples (Figures 2C,D). Further, the composition of the microbiota differed between samples collected from different sites in January. The dominant genera in samples from site A were *Pseudomonas*, *Carnobacterium*, and *Brochothrix*, while the dominant genera in samples collected from site B were *Serratia*, *Kocuria*, and *Corynebacterium* and in those collected from site C, *Escherichia*, *Macrococcus*, and *Salmonella*. In July samples, *Carnobacterium* (average 28.11%), *Lactobacillus* (average 19.49%), and *Pseudomonas* (average 14.54%) were dominant in all samples. However, *Serratia* and *Kocuria* were dominant only in samples of site B, like the microbiota in January samples. *Carnobacterium* is a prevalent member of lactic acid bacteria (LAB) in fresh meat and processed meat products (Pothakos et al., 2015). Psychrotrophic bacteria such as LAB and *Pseudomonas* spp. can easily dominate in meat products stored at under chilled conditions (Stanborough et al., 2017). Although the average temperature in July at the three sites was above 26°C (27.2°C at site A, 28.3°C at site B, 26.7°C at site C), psychrotrophic bacteria were dominant in July. This could be because of the cold temperatures (below 10°C) during processing in the LPC and during transportation conditions. Though the cold temperature in the LPC and transportation was also maintained in January, the more diverse microbiota in January could be because of the storage duration of beef meat. An earlier study has reported higher bacterial diversity in fresh-cut beef than the later stage of storage (Säde et al., 2017).

In addition, the dominant genera differed by sampling sites. *Serratia* was especially dominant in ground beef from site B in both months (26.32% in January and 24.0% in July). *Serratia* spp. is commonly found in meat (Doulgeraki et al., 2011) and is also known as a major spoilage *Enterobacteriaceae* (Ercolini et al., 2006). However, *Escherichia* (16.32% in ground beef) and *Salmonella* (15.22% in non-ground beef) were dominant in beef samples from site C in January. The observed differences of microbiota could be because of the environments of the LPC, farm, and individual differences of microbiota in cattle. Cattle from different farms were gathered to the LPC of each site; thus, the microbiota in cattle was already different before slaughter. However, cattle were washed and processed under a controlled environment in the LPC. Therefore, the microbiota in beef meat could be influenced more by the environment of the LPC, implicating its importance for food safety. The findings of this study were supported by earlier studies, showing the importance of the processing environment for food safety and reducing microbial contamination (Rivera-Betancourt et al., 2004; Nychas et al., 2008; Stellato et al., 2016).

### Factors Influencing the Differences in Microbiota in Different Beef Samples

**FIGURE 3:**
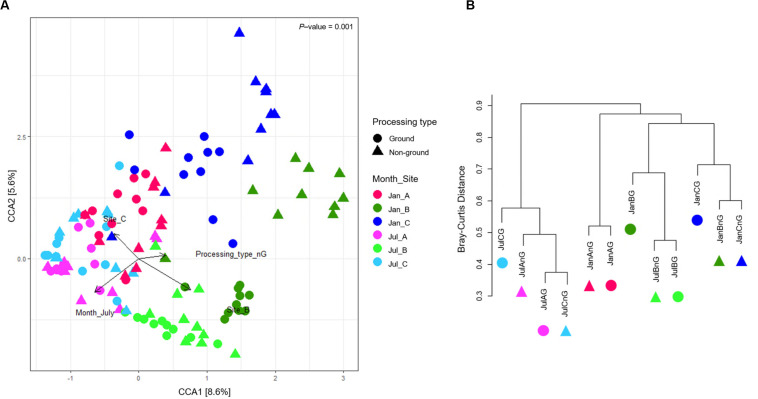
The dissimilarity of microbiota in beef meat samples. **(A)** Canonical correspondence analysis (CCA) biplot of beef microbiota. The points indicate the microbiota of each sample, and the dissimilarity was calculated by Bray-Curtis distance. The axes represent the percentage of the corresponding total variance. The variables (sampling months, sites, and processing types) were depicted as vectors. The longer vector has stronger effects on the dispersion of microbiota. **(B)** Clustering of microbiota based on Bray-Curtis distance between samples.

Canonical correspondence analysis was used to show the correlation of microbiota difference with environmental variables (Figure 3A). The total inertia of the CCA plot was 5.88, and the constrained inertia was 1.13. A total of 8.6% of the constrained inertia was explained by the CCA1 axis, while CCA2 explained a further 5.6%. Arrows on the plot showed the strength of the plot dispersion. Among the arrows, sampling sites and months had a more significant influence on microbiota dispersion than the processing type. Microbiota in January samples were more distinguished according to sampling sites than those in July samples. For July, samples from site B were significantly different from the other two sites. This difference was also observed in the genera composition of samples from site B (Figure 2D). We found that beef microbiota were significantly different in the ground samples from site B and C in January. In addition, the cluster dendrogram also showed that the distance between ground beef and non-ground beef was relatively high in these sites, while other samples shared similar communities irrespective of the processing types (Figure 3B). The CCA plot indicated that the regional and seasonal factors comprehensively affected the diversity of beef microbiota. Earlier studies have shown the possibility of transmission of the microbes present in beef, cattle, and the processing machinery to the beef products (Elder et al., 2000; Stellato et al., 2016). From the present findings, we also speculated that the cleanliness of farm and processing environments, water quality, and storage conditions of the LPC could have influenced the composition of the microbiota in the beef products. Hence, there is a need for careful process management before, during, and after slaughtering.

The genera in the samples collected from different sites at different months subjected to different grinding processes were determined by DeSeq2 based on the log2 fold change values of genus proportion (Supplementary Table S2). Average of 14 genera for non-ground and 15 genera for ground beef were found to be significantly different between sampling months in each sampling site (FDR < 0.01). *Propionibacterium* was dominant in January samples from all sites (5.62 ± 0.70-fold), while *Serratia* (4.71 ± 0.27-fold), *Hafnia* (6.39 ± 0.77–fold), *Lactobacillus* (5.26 ± 0.25–fold), and *Lactococcus* (12.42 ± 7.46-fold) were dominant in July samples. The dominance of *Propionibacterium* in January samples could be because of contamination from human skin and indoor slaughter environments (Jeon et al., 2013; Stoops et al., 2015; Alessandria et al., 2016). However, a reduced level of *Propionibacterium* in the July sample could be for the dominance of LAB, such as *Lactobacillus* and *Lactococcus*. Reported earlier, these LAB could inhibit the growth of *Propionibacterium acnes* through the secretion of bacteriocins (Oh et al., 2006; Kober and Bowe, 2015). In addition, LAB play a major role in beef spoilage even at refrigerated temperatures (Nychas and Skandamis, 2005), and the abundance of LAB, *Serratia*, and *Hafnia* genera in the present study could be related to the beef spoilage. These results indicated that the environmental conditions in July favor spoilage; hence, management of beef meat should be paramount in July for reducing risk of spoilage.

The ground samples collected from the three sites in January and July identified 7–19 significantly different genera (FDR < 0.01), however, we did not observe any significant difference between the ground and non-ground beef from sites A and C in July (FDR > 0.01). In addition, the cluster dendrogram showed that the similarity of microbiota between ground and non-ground beef from the same sites in each month was relatively high (Figure 3B). The findings of this study indicated that the ground process is not a factor influencing the microbiota in beef meat.

### The Core Genera in Beef Microbiota in All Samples

Based on the results of the cluster dendrogram that showed the minimal influence of the ground process on the community dissimilarity (Figure 3B), we determined the core genera in the beef samples collected from different sampling sites and times (Supplementary Figure S2). The identified core genera are summarized in Supplementary Table S3. A total of 52 genera were detected as core genera among all beef samples with 27 genera in January and 25 genera in July samples.

**FIGURE 4:**
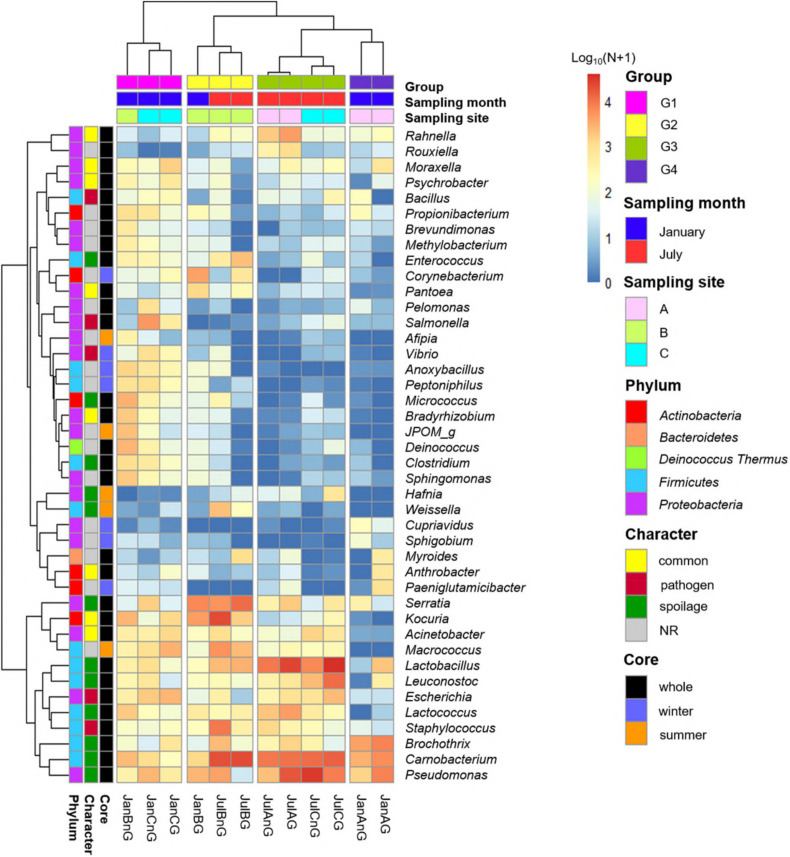
Comparison of core genera using heat map analysis. The relative abundances of genera were calculated by a log10 scale. Samples were clustered to groups 1, 2, 3, and 4 by spearman correlations. Core genera were characterized by common, pathogen, spoilage, and NR.

Subsequently, the relative abundances of core genera were compared among samples through heat map analysis (Figure 4). The samples were clustered into four groups based on Spearman correlation, and groups were distinguished by the sampling month (January samples in groups 1 and 4; July samples in groups 2 and 3). We then classified the genera into four character groups related to potential risk factors according to the previous studies (Nychas et al., 2008; Doulgeraki et al., 2012; Iulietto et al., 2015). The groups were characterized as common (commonly found genera in fresh beef), spoilage (genera related to beef spoilage), pathogen (related to potential foodborne pathogens), and NR (not reported). We identified 8 genera as common, 12 genera as spoilage, 5 genera as pathogen, and 17 genera as NR (Table 1). In this study, the relative abundance of spoilage genera (63.11%) in beef microbiota was highest (*p* < 0.001), followed by NR (12.61%), common (11.17%), and pathogen (7.71%). Still, 84.59% of the core genera were *Firmicutes* and *Proteobacteria* and mostly comprised the spoilage group that included 8 genera of *Firmicutes*, 3 genera of *Proteobacteria*, and one genus of *Actinobacteria*. These results were consistent with previous studies, which have reported *Firmicutes* and *Proteobacteria* as the dominant phyla in spoiled beef (Vihavainen and Björkroth, 2007; De Filippis et al., 2013).

**TABLE 1:** Core genera in the microbiota of beef meat. Genera were classified into four character groups according to the previous studies (Nychas et al., 2008; Doulgeraki et al., 2012; Iulietto et al., 2015).

	Genus	Phylum	Character	Mean abundance ± *SD* (%)
Core genera in all samples	*Acinetobacter*	*Proteobacteria*	Common	1.53 ± 1.57
	*Arthrobacter*	*Actinobacteria*	Common	0.30 ± 0.72
	*Bacillus*	*Firmicutes*	Pathogen	0.46 ± 0.58
	*Bradyrhizobium*	*Proteobacteria*	Common	0.65 ± 1.43
	*Brevundimonas*	*Proteobacteria*	Not reported	0.23 ± 0.45
	*Brochothrix*	*Firmicutes*	Spoilage	5.17 ± 8.10
	*Carnobacterium*	*Firmicutes*	Spoilage	20.37 ± 13.33
	*Clostridium*	*Firmicutes*	Spoilage	0.66 ± 1.22
	*Deinococcus*	*Deinococcus Thermus*	Not reported	0.94 ± 2.16
	*Enterococcus*	*Firmicutes*	Spoilage	0.79 ± 1.27
	*Escherichia*	*Proteobacteria*	Pathogen	3.36 ± 4.51
	*Kocuria*	*Actinobacteria*	Common	5.10 ± 8.00
	*Lactobacillus*	*Firmicutes*	Spoilage	10.85 ± 12.29
	*Lactococcus*	*Firmicutes*	Spoilage	2.03 ± 2.16
	*Leuconostoc*	*Firmicutes*	Spoilage	2.11 ± 3.02
	*Methylobacterium*	*Proteobacteria*	Not reported	0.26 ± 0.42
	*Micrococcus*	*Actinobacteria*	Spoilage	1.07 ± 2.36
	*Moraxella*	*Proteobacteria*	Common	1.16 ± 1.62
	*Myroides*	*Bacteroidetes*	Not reported	0.39 ± 0.77
	*Pantoea*	*Proteobacteria*	Common	0.53 ± 1.40
	*Pelomonas*	*Proteobacteria*	Not reported	0.54 ± 1.52
	*Propionibacterium*	*Actinobacteria*	Not reported	1.12 ± 1.45
	*Pseudomonas*	*Proteobacteria*	Spoilage	13.93 ± 10.51
	*Psychrobacter*	*Proteobacteria*	Common	0.59 ± 1.01
	*Rahnella*	*Proteobacteria*	Common	1.31 ± 2.21
	*Rouxiella*	*Proteobacteria*	Not reported	0.29 ± 0.53
	*Salmonella*	*Proteobacteria*	Pathogen	1.48 ± 4.18
	*Serratia*	*Proteobacteria*	Spoilage	5.65 ± 8.92
	*Sphingomonas*	*Proteobacteria*	Not reported	0.65 ± 1.40
	*Staphylococcus*	*Firmicutes*	Pathogen	1.98 ± 2.85
Core genera in January samples	*Anoxybacillus*	*Firmicutes*	Not reported	1.02 ± 1.66
	*Corynebacterium*	*Actinobacteria*	Not reported	1.67 ± 4.51
	*Cupriavidus*	*Proteobacteria*	Not reported	0.17 ± 0.45
	*Paeniglutamicibacter*	*Actinobacteria*	Not reported	0.37 ± 1.05
	*Peptoniphilus*	*Firmicutes*	Not reported	0.90 ± 1.70
	*Sphingobium*	*Proteobacteria*	Not reported	0.12 ± 0.31
	*Vibrio*	*Proteobacteria*	Pathogen	0.44 ± 0.89
Core genera in July samples	*Afipia*	*Proteobacteria*	Not reported	0.13 ± 0.34
	*Hafnia*	*Proteobacteria*	Spoilage	0.17 ± 0.44
	*JPOM_g*	*Proteobacteria*	Not reported	0.69 ± 1.88
	*Macrococcus*	*Firmicutes*	Not reported	3.12 ± 4.01
	*Weissella*	*Firmicutes*	Spoilage	0.30 ± 0.83

*Common, commonly found genus in fresh beef; Spoilage, genus related to beef spoilage; Pathogen, genus related to potential foodborne pathogens; Not reported, not reported genus in beef meat.*

The genus *Salmonella* belonging to the group “pathogen” was found to be more abundant in beef samples from site C in January than the other three groups. It has been shown that *Salmonella* spp. are the most common pathogens causing foodborne illness related to beef consumption (Dechet et al., 2006; Laufer et al., 2015). A previous study reported that *S. enterica* was more abundant in final beef products than in the feedlot (Yang et al., 2016). Although various pathogens can be eliminated by the application of surface antimicrobial treatments, *Salmonella* spp. can survive by internalization into peripheral lymph nodes and multiply (Brichta-Harhay et al., 2012).

The relative abundances of genera related to spoilage were higher in the samples of group 2, 3, and 4 (60.84, 82.82, and 84.90% of core genera, respectively). The risk of spoilage might be higher in the beef samples of these groups. Although the relative abundances of *Carnobacterium* and *Pseudomonas* were high in these beef samples, the proportions of spoilage genera were different among samples in these groups. *Serratia* was the dominant genus in group 2 (19.05%), *Lactobacillus* in group 3 (27.05%), and *Brochothrix* in group 4 (22.86%). These differences could be because of the existence of different microbiota in different samples and the influence of the environmental conditions of the respective LPC. The findings of this study reveal that the composition of microbiota in beef meat could provide the information for microbial risks related to spoilage.

### The Predicted Function of the Beef Core Microbiota

**FIGURE 5:**
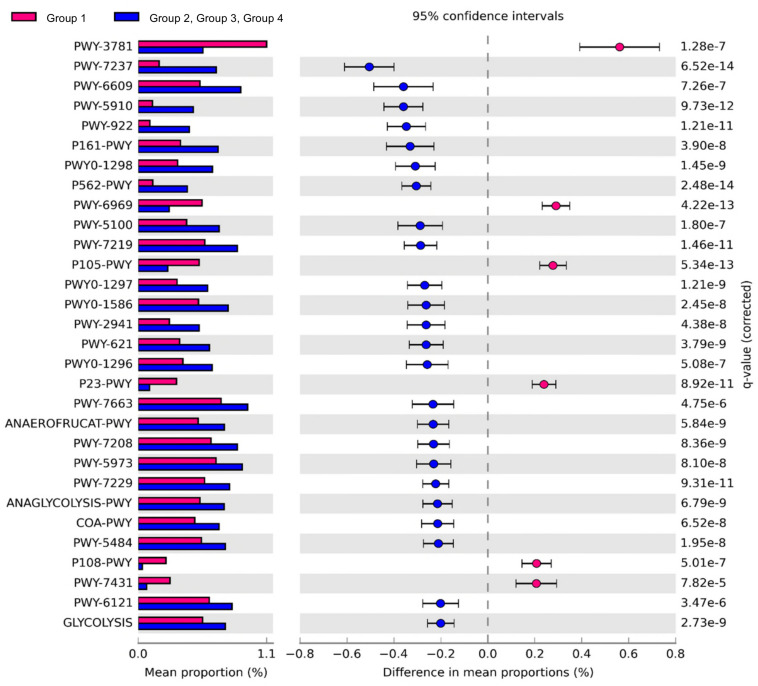
Comparison of predicted pathways between group 1 and the other groups clustered in heat map analysis (Figure 4). Pathways were predicted by PICRUSt2. The significantly different pathways between groups (Welch’s *t*-test *q* < 0.01) are shown.

Comparison of the predicted functions of microbiota between samples in group 1 and other groups using PICRUSt2 (Figure 5) identified 221 significantly different pathways between group 1 and other groups (FDR < 0.01). The details of the groups with over 0.20% difference are provided in Figure 5 and Supplementary Table S4.

The pathways related to the TCA cycle and aerobic respiration were significantly prominent in the samples of group 1 than in the other groups (Supplementary Table S4). These results indicated that the aerobic bacteria were predominant and played important roles in the samples of group 1. We also observed higher abundance of *Salmonella* in these samples. The results were consistent with a previous study that showed the abundance of *Salmonella* corresponded to the abundance of aerobic bacteria in beef (Brichta-Harhay et al., 2008). Here, the proportions of pathways related to fermentation and glycolysis were significantly higher in samples of groups 2, 3, and 4. These results indicated that fermentation may be the key pathway leading to beef spoilage. Homolactic fermentation (ANAEROFRUCAT-PWY) and pyruvate fermentation to acetate and lactate II (PWY-5100) pathways were significantly higher in the samples of groups 2, 3, and 4 than in the samples of group 1. These pathways were also related to beef souring in a previous study (Nychas et al., 2008). Lactate can maintain a low pH in beef meat; thus, bacteria with acid-tolerance can still thrive and dominate in such environment (Alvarado and McKee, 2007; Kalchayanand et al., 2018). The proportions of glycolysis and sucrose degradation pathways were significantly higher in the samples of groups 2, 3, and 4. Glucose is one of the main precursors for off-flavors and acids by spoilage microbes such as *Carnobacterium, Brochothrix*, LAB, and *Pseudomonas* (Gram et al., 2002). In addition, *Lactobacillus* can cause severe acidification in beef and emission of off-odor compounds, while *Leuconostoc* can produce organic acid such as acetic acid by using glucose in spoiled beef (Samelis et al., 2006; Doulgeraki et al., 2010; Pothakos et al., 2015). Therefore, spoilage genera in the samples of groups 2, 3, and 4 could dominate by producing acid and, as a result, could be related to beef spoilage in beef meat.

Microbiota in samples of group 1 were more diverse than those of other groups, and the relative abundances of aerobic bacteria were higher in the microbiota of group 1. In contrast to microbiota in the samples of group 1, the microbes related to the spoilage were more detected in the microbiota of groups 2, 3, and 4 samples, even at cold temperatures. These bacteria may inhibit the aerobic bacteria through fermentation in beef meat. Therefore, beef meat may be spoiled. The initial contaminating microbes and the storage condition were important to the later stage microbiota in beef (Hilgarth et al., 2018), and spoilage could occur by metabolites produced by spoilage microbes (Jääskeläinen et al., 2016). In this study, the dominance of spoilage microbes may be related to pathways of glucose utilization, and it could cause beef meat spoilage by resident microbiota.

In this study, we analyzed the microbiota in fresh beef meat and their potential functions by microbiota characteristics. The microbiota in fresh beef meat differed according to sampling sites and months, but core genera were detected in all samples. The potential spoilage genera were prominent in fresh beef meat, and these genera could influence the growth of other microbes using glucose by fermentation. Beef meat has a glucose-enriched environment; thus, strategies to inhibit the spoilage microbes using obtained information can reduce and prevent the spoilage of beef meat by microbiota. Although further studies such as co-culturing, metatranscriptomic, and metabolomics analyses are necessary to validate the results, the findings in this study provide information on initial microbiota to understand the bacterial risk of spoilage in beef meat products.

## Data Availability Statement

All sequences were deposited on European Nucleotide Archive (ENA) study accession number PRJEB35021 (https://www.ebi.ac.uk/ena/data/search?query=PRJEB35021).

## Author Contributions

SC and B-SK: conception and design. BH and HC: acquisition of data. BH: data analysis and writing of the draft manuscript. BH and B-SK: data interpretation. BH, HC, SC, and B-SK: review and editing the manuscript. All authors contributed to the article and approved the submitted version.

## Conflict of Interest

The authors declare that the research was conducted in the absence of any commercial or financial relationships that could be construed as a potential conflict of interest.

## Footnotes

1 http://www.ekapepia.com

2 http://www.drive5.com/usearch
