# Supplementary material for: Analysis of Microbiota Structure and Potential Functions Influencing Spoilage of Fresh Beef Meat
Source: Front Microbiol. 2020 Jul 22;11:1657. doi: 10.3389/fmicb.2020.01657 (PMC7387507; doi:10.3389/fmicb.2020.01657)
Supplement: Supplementary file 1 [file Data_Sheet_1.docx]

***Supplementary Materials***

**Analysis of Microbiota Structure and Potential Functions Influencing Spoilage of Fresh Beef Meat**

Bo Kyoung Hwang^1^, HyeLim Choi^1^, Sang Ho Choi^1,*^, and Bong-Soo Kim^2,*^

^1^Department of Agricultural Biotechnology, Center of Food Safety and Toxicology, Seoul National University, Seoul, South Korea

^2^Department of Life Science, Multidisciplinary Genome Institute, Hallym University, Chuncheon, South Korea

| **Supplemenatary Table S1.** Summary of diversity indices obtained through Illumina MiSeq sequencing | | | | | |  |
| --- | --- | --- | --- | --- | --- | --- |
| **Beef Samples** | | | | **Number of reads** | **Observed OTUs** | **Shannon diversity index** |
| **Sampling month** | **Sampling sites** | **Processing types** | **Sample** |  |  |  |
| **January** | **A** | **non-ground** | JanAnG_1 | 11,002 | 6,764 | 3.43 |
|  |  |  | JanAnG_2 | 2,756 | 1,682 | 3.19 |
|  |  |  | JanAnG_3 | 3,488 | 2,219 | 2.91 |
|  |  |  | JanAnG_4 | 4,428 | 2,877 | 2.23 |
|  |  |  | JanAnG_5 | 6,559 | 4,625 | 1.80 |
|  |  |  | JanAnG_6 | 21,621 | 10,832 | 3.61 |
|  |  |  | JanAnG_7 | 36,045 | 23,475 | 2.46 |
|  |  |  | JanAnG_8 | 37,394 | 28,127 | 1.14 |
|  |  |  | JanAnG_9 | 28,054 | 19,046 | 2.76 |
|  |  |  | JanAnG_10 | 20,173 | 14,507 | 1.86 |
|  |  | **ground** | JanAG_1 | 58,798 | 37,871 | 3.11 |
|  |  |  | JanAG_2 | 25,210 | 16,988 | 3.34 |
|  |  |  | JanAG_3 | 40,406 | 23,095 | 3.95 |
|  |  |  | JanAG_4 | 75,094 | 47,382 | 3.71 |
|  |  |  | JanAG_5 | 45,096 | 29,790 | 2.91 |
|  |  |  | JanAG_6 | 17,492 | 11,696 | 3.55 |
|  |  |  | JanAG_7 | 48,658 | 34,240 | 2.87 |
|  |  |  | JanAG_8 | 55,890 | 37,180 | 2.62 |
|  |  |  | JanAG_9 | 56,688 | 37,160 | 2.52 |
|  |  |  | JanAG_10 | 17,870 | 11,538 | 2.33 |
|  | **B** | **non-ground** | JanBnG_1 | 40,354 | 22,723 | 4.32 |
|  |  |  | JanBnG_2 | 71,675 | 37,355 | 4.55 |
|  |  |  | JanBnG_3 | 68,525 | 36,607 | 4.19 |
|  |  |  | JanBnG_4 | 78,700 | 49,386 | 3.97 |
|  |  |  | JanBnG_5 | 60,090 | 34,905 | 4.45 |
|  |  |  | JanBnG_6 | 69,832 | 35,807 | 4.75 |
|  |  |  | JanBnG_7 | 72,384 | 37,706 | 4.42 |
|  |  |  | JanBnG_8 | 61,493 | 32,607 | 4.78 |
|  |  |  | JanBnG_9 | 71,112 | 39,261 | 4.61 |
|  |  |  | JanBnG_10 | 38,460 | 21,963 | 4.35 |
|  |  | **ground** | JanBG_1 | 51,560 | 26,124 | 4.17 |
|  |  |  | JanBG_2 | 42,104 | 20,252 | 4.17 |
|  |  |  | JanBG_3 | 63,307 | 31,922 | 4.30 |
|  |  |  | JanBG_4 | 53,126 | 28,627 | 4.22 |
|  |  |  | JanBG_5 | 43,455 | 24,321 | 4.19 |
|  |  |  | JanBG_6 | 39,228 | 20,702 | 4.17 |
|  |  |  | JanBG_7 | 56,868 | 31,746 | 4.15 |
|  |  |  | JanBG_8 | 41,397 | 22,022 | 4.59 |
|  |  |  | JanBG_9 | 42,078 | 23,404 | 4.06 |
|  |  |  | JanBG_10 | 57,698 | 27,163 | 4.20 |
|  | **C** | **non-ground** | JanCnG_1 | 40,750 | 22,153 | 4.57 |
|  |  |  | JanCnG_2 | 83,480 | 54,084 | 2.63 |
|  |  |  | JanCnG_3 | 79,527 | 48,874 | 3.09 |
|  |  |  | JanCnG_4 | 30,098 | 17,426 | 3.79 |
|  |  |  | JanCnG_5 | 57,539 | 27,364 | 4.52 |
|  |  |  | JanCnG_6 | 78,456 | 43,836 | 4.05 |
|  |  |  | JanCnG_7 | 4,755 | 2,473 | 4.02 |
|  |  |  | JanCnG_8 | 5,486 | 3,454 | 3.51 |
|  |  |  | JanCnG_9 | 4,283 | 2,549 | 3.35 |
|  |  |  | JanCnG_10 | 65,501 | 49,745 | 3.25 |
|  |  | **ground** | JanCG_1 | 47,421 | 26,459 | 4.94 |
|  |  |  | JanCG_2 | 2,718 | 1,735 | 3.50 |
|  |  |  | JanCG_3 | 24,522 | 15,021 | 1.17 |
|  |  |  | JanCG_4 | 2,145 | 1,260 | 4.01 |
|  |  |  | JanCG_5 | 74,030 | 51,046 | 3.82 |
|  |  |  | JanCG_6 | 66,660 | 44,849 | 4.24 |
|  |  |  | JanCG_7 | 46,884 | 32,633 | 4.41 |
|  |  |  | JanCG_8 | 12,918 | 9,118 | 4.25 |
|  |  |  | JanCG_9 | 22,064 | 12,105 | 3.60 |
|  |  |  | JanCG_10 | 3,083 | 1,663 | 4.22 |
| **July** | **A** | **non-ground** | JulAnG_1 | 41,054 | 30,699 | 1.91 |
|  |  |  | JulAnG_2 | 121,269 | 71,952 | 3.83 |
|  |  |  | JulAnG_3 | 31,251 | 18,612 | 3.69 |
|  |  |  | JulAnG_4 | 66,482 | 46,824 | 2.73 |
|  |  |  | JulAnG_5 | 38,782 | 23,487 | 3.74 |
|  |  |  | JulAnG_6 | 17,792 | 9,705 | 3.86 |
|  |  |  | JulAnG_7 | 15,068 | 8,375 | 3.85 |
|  |  |  | JulAnG_8 | 62,453 | 38,997 | 2.59 |
|  |  |  | JulAnG_9 | 62,068 | 38,306 | 2.55 |
|  |  |  | JulAnG_10 | 24,688 | 16,618 | 3.25 |
|  |  | **ground** | JulAG_1 | 51,116 | 29,599 | 3.74 |
|  |  |  | JulAG_2 | 94,347 | 63,311 | 2.98 |
|  |  |  | JulAG_3 | 119,727 | 75,567 | 3.44 |
|  |  |  | JulAG_4 | 92,037 | 69,458 | 2.11 |
|  |  |  | JulAG_5 | 150,001 | 101,813 | 2.95 |
|  |  |  | JulAG_6 | 82,136 | 65,386 | 2.45 |
|  |  |  | JulAG_7 | 94,703 | 68,842 | 3.42 |
|  |  |  | JulAG_8 | 208,301 | 157,294 | 2.56 |
|  |  |  | JulAG_9 | 81,371 | 58,932 | 2.84 |
|  |  |  | JulAG_10 | 23,098 | 16,732 | 3.17 |
|  | **B** | **non-ground** | JulBnG_1 | 65,485 | 47,749 | 3.29 |
|  |  |  | JulBnG_2 | 72,001 | 55,706 | 3.23 |
|  |  |  | JulBnG_3 | 94,589 | 74,712 | 2.95 |
|  |  |  | JulBnG_4 | 76,867 | 60,019 | 3.03 |
|  |  |  | JulBnG_5 | 284,448 | 224,623 | 2.77 |
|  |  |  | JulBnG_6 | 147,746 | 90,190 | 2.57 |
|  |  |  | JulBnG_7 | 83,251 | 57,559 | 3.08 |
|  |  |  | JulBnG_8 | 68,240 | 44,990 | 3.68 |
|  |  |  | JulBnG_9 | 130,315 | 84,363 | 2.85 |
|  |  |  | JulBnG_10 | 85,197 | 48,967 | 3.96 |
|  |  | **ground** | JulBG_1 | 77,165 | 59,508 | 1.47 |
|  |  |  | JulBG_2 | 58,936 | 46,785 | 2.75 |
|  |  |  | JulBG_3 | 62,419 | 48,154 | 2.69 |
|  |  |  | JulBG_4 | 54,512 | 40,571 | 2.16 |
|  |  |  | JulBG_5 | 78,264 | 57,646 | 2.43 |
|  |  |  | JulBG_6 | 84,533 | 47,851 | 3.87 |
|  |  |  | JulBG_7 | 71,697 | 44,462 | 2.98 |
|  |  |  | JulBG_8 | 69,961 | 41,172 | 3.12 |
|  |  |  | JulBG_9 | 64,539 | 43,614 | 2.46 |
|  |  |  | JulBG_10 | 104,940 | 65,482 | 2.85 |
|  | **C** | **non-ground** | JulCnG_1 | 63,362 | 48,852 | 2.21 |
|  |  |  | JulCnG_2 | 37,084 | 28,292 | 1.96 |
|  |  |  | JulCnG_3 | 117,181 | 97,471 | 1.42 |
|  |  |  | JulCnG_4 | 189,701 | 165,129 | 0.95 |
|  |  |  | JulCnG_5 | 64,503 | 31,491 | 4.25 |
|  |  |  | JulCnG_6 | 69,395 | 60,974 | 0.17 |
|  |  |  | JulCnG_7 | 18,923 | 15,698 | 2.62 |
|  |  |  | JulCnG_8 | 39,451 | 33,492 | 2.18 |
|  |  |  | JulCnG_9 | 25,682 | 22,449 | 1.97 |
|  |  |  | JulCnG_10 | 64,527 | 52,197 | 2.11 |
|  |  | **ground** | JulCG_1 | 65,096 | 54,161 | 1.11 |
|  |  |  | JulCG_2 | 516,550 | 430,355 | 1.79 |
|  |  |  | JulCG_3 | 91,631 | 60,374 | 2.74 |
|  |  |  | JulCG_4 | 84,320 | 51,077 | 3.16 |
|  |  |  | JulCG_5 | 32,515 | 20,707 | 2.67 |
|  |  |  | JulCG_6 | 85,777 | 44,396 | 4.15 |
|  |  |  | JulCG_7 | 36,351 | 26,462 | 2.30 |
|  |  |  | JulCG_8 | 47,299 | 40,601 | 2.04 |
|  |  |  | JulCG_9 | 63,733 | 53,621 | 1.83 |
|  |  |  | JulCG_10 | 37,001 | 30,002 | 2.19 |

| **Supplementary Table S2.** The list of log2 fold change value according to sampling months and processing types | | | | | |
| --- | --- | --- | --- | --- | --- |
| **Seasonal** | | | | | |
| **Location** | **Processing type** | **Increasing category** | **Genus** | **log2FoldChange** | **padj** |
| **Location A** | Non-ground beef | January | *Brevundimonas* | 7.51 | 5.E-04 |
|  |  |  | *Propionibacterium* | 6.45 | 2.E-04 |
|  |  |  | *Pelomonas* | 5.78 | 8.E-03 |
|  |  |  | *Brochothrix* | 5.28 | 9.E-08 |
|  |  | July | *Serratia* | -4.44 | 1.E-03 |
|  |  |  | *Acinetobacter* | -5.44 | 4.E-03 |
|  |  |  | *Hafnia* | -5.79 | 1.E-03 |
|  |  |  | *Leuconostoc* | -8.19 | 2.E-04 |
|  |  |  | *Lactobacillus* | -8.93 | 4.E-09 |
|  |  |  | *Lactococcus* | -27.07 | 2.E-19 |
|  | Ground beef | January | *Arthrobacter* | 5.75 | 1.E-10 |
|  |  |  | *Myroides* | 4.46 | 7.E-05 |
|  |  |  | *Brochothrix* | 4.25 | 5.E-11 |
|  |  |  | *Moraxella* | 3.23 | 5.E-03 |
|  |  | July | *Lactobacillus* | -2.09 | 6.E-04 |
|  |  |  | *Rahnella* | -2.85 | 3.E-06 |
|  |  |  | *Rouxiella* | -3.39 | 2.E-07 |
|  |  |  | *Serratia* | -4.70 | 4.E-09 |
|  |  |  | *Hafnia* | -6.66 | 6.E-07 |
|  |  |  | *Lactococcus* | -7.56 | 6.E-33 |
|  |  |  | *Macrococcus* | -8.36 | 1.E-04 |
| **Location B** | Non-ground beef | January | *Peptoniphilus* | 7.38 | 1.E-05 |
|  |  |  | *JPOM_g* | 6.53 | 5.E-06 |
|  |  |  | *Afipia* | 6.26 | 1.E-05 |
|  |  |  | *Deinococcus* | 5.87 | 1.E-05 |
|  |  |  | *Clostridium* | 5.55 | 4.E-05 |
|  |  |  | *Brevundimonas* | 5.36 | 5.E-06 |
|  |  |  | *Sphingomonas* | 5.09 | 7.E-06 |
|  |  |  | *Bradyrhizobium* | 4.91 | 9.E-05 |
|  |  |  | *Propionibacterium* | 3.51 | 2.E-03 |
|  |  | July | *Lactobacillus* | -2.81 | 6.E-03 |
|  |  |  | *Kocuria* | -3.53 | 5.E-05 |
|  |  |  | *Carnobacterium* | -3.68 | 2.E-05 |
|  |  |  | *Serratia* | -4.23 | 1.E-05 |
|  |  |  | *Brochothrix* | -4.74 | 4.E-11 |
|  |  |  | *Macrococcus* | -5.23 | 5.E-09 |
|  |  |  | *Staphylococcus* | -5.97 | 1.E-16 |
|  |  |  | *Weissella* | -10.78 | 3.E-20 |
|  | Ground beef | January | *Anoxybacillus* | 8.33 | 2.E-13 |
|  |  |  | *Sphingomonas* | 8.08 | 4.E-14 |
|  |  |  | *Clostridium* | 7.81 | 4.E-14 |
|  |  |  | *Methylobacterium* | 7.35 | 4.E-11 |
|  |  |  | *Brevundimonas* | 6.46 | 1.E-08 |
|  |  |  | *JPOM_g* | 6.43 | 6.E-12 |
|  |  |  | *Pseudomonas* | 6.41 | 3.E-11 |
|  |  |  | *Deinococcus* | 6.37 | 3.E-10 |
|  |  |  | *Propionibacterium* | 6.22 | 5.E-11 |
|  |  |  | *Bradyrhizobium* | 5.67 | 7.E-08 |
|  |  |  | *Arthrobacter* | 4.77 | 5.E-06 |
|  |  |  | *Psychrobacter* | 4.74 | 3.E-07 |
|  |  |  | *Afipia* | 4.07 | 6.E-03 |
|  |  |  | *Moraxella* | 3.07 | 4.E-03 |
|  |  | July | *Myroides* | -3.01 | 8.E-03 |
|  |  |  | *Lactobacillus* | -4.25 | 2.E-07 |
|  |  |  | *Carnobacterium* | -4.62 | 9.E-17 |
|  |  |  | *Hafnia* | -4.94 | 1.E-09 |
|  |  |  | *Macrococcus* | -5.36 | 1.E-08 |
|  |  |  | *Enterococcus* | -5.92 | 2.E-13 |
|  |  |  | *Weissella* | -6.95 | 1.E-09 |
| **Location C** | Non-ground beef | January | *Anoxybacillus* | 10.01 | 1.E-17 |
|  |  |  | *Peptoniphilus* | 8.25 | 1.E-14 |
|  |  |  | *Pelomonas* | 6.69 | 1.E-07 |
|  |  |  | *Salmonella* | 6.53 | 1.E-06 |
|  |  |  | *Propionibacterium* | 6.29 | 3.E-09 |
|  |  |  | *Vibrio* | 5.94 | 2.E-05 |
|  |  |  | *Clostridium* | 5.62 | 6.E-08 |
|  |  |  | *Deinococcus* | 4.74 | 3.E-06 |
|  |  |  | *Sphingomonas* | 3.23 | 4.E-03 |
|  |  | July | *Acinetobacter* | -3.44 | 3.E-03 |
|  |  |  | *Pseudomonas* | -4.11 | 1.E-03 |
|  |  |  | *Lactobacillus* | -4.31 | 4.E-03 |
|  |  |  | *Brochothrix* | -4.99 | 2.E-04 |
|  |  |  | *Rahnella* | -5.06 | 4.E-03 |
|  |  |  | *Carnobacterium* | -5.17 | 2.E-03 |
|  | Ground beef | January | *Anoxybacillus* | 9.09 | 2.E-11 |
|  |  |  | *Peptoniphilus* | 7.18 | 2.E-07 |
|  |  |  | *Clostridium* | 5.33 | 1.E-03 |
|  |  |  | *Micrococcus* | 5.26 | 5.E-04 |
|  |  |  | *Vibrio* | 4.84 | 1.E-03 |
|  |  |  | *Psychrobacter* | 3.66 | 9.E-03 |
|  |  | July | *Lactococcus* | -2.62 | 7.E-03 |
|  |  |  | *Pseudomonas* | -4.43 | 3.E-06 |
|  |  |  | *Rouxiella* | -5.39 | 2.E-04 |
|  |  |  | *Serratia* | -5.48 | 3.E-04 |
|  |  |  | *Hafnia* | -7.57 | 2.E-06 |
|  |  |  | *Carnobacterium* | -7.70 | 2.E-14 |
|  |  |  | *Lactobacillus* | -9.19 | 5.E-15 |
|  |  |  | *Leuconostoc* | -9.43 | 7.E-15 |
| **Processing type** | | | | | |
| **Location** | **Season** | **Increasing category** | **Genus** | **log2FoldChange** | **padj** |
| **Location A** | January | Non-ground beef | *Enterococcus* | 7.16 | 2.E-06 |
|  |  |  | *Propionibacterium* | 3.05 | 9.E-04 |
|  |  | Ground beef | *Moraxella* | -3.91 | 2.E-04 |
|  |  |  | *Lactococcus* | -4.43 | 4.E-03 |
|  |  |  | *Psychrobacter* | -4.57 | 1.E-05 |
|  |  |  | *Lactobacillus* | -7.03 | 3.E-11 |
|  |  |  | *Leuconostoc* | -9.30 | 2.E-26 |
|  |  |  | *Myroides* | -10.16 | 6.E-24 |
|  |  |  | *Arthrobacter* | -10.55 | 9.E-31 |
|  | July |  | *N.D** | | |
| **Location B** | January | Non-ground beef | *JPOM_g* | 4.86 | 2.E-10 |
|  |  |  | *Vibrio* | 4.75 | 5.E-03 |
|  |  |  | *Afipia* | 4.43 | 2.E-06 |
|  |  |  | *Micrococcus* | 4.11 | 2.E-10 |
|  |  |  | *Deinococcus* | 3.75 | 1.E-08 |
|  |  |  | *Bacillus* | 3.44 | 3.E-03 |
|  |  |  | *Bradyrhizobium* | 3.39 | 1.E-07 |
|  |  |  | *Brevundimonas* | 2.71 | 2.E-03 |
|  |  |  | *Sphingomonas* | 2.64 | 6.E-05 |
|  |  |  | *Clostridium* | 2.06 | 1.E-03 |
|  |  |  | *Enterococcus* | 1.37 | 3.E-03 |
|  |  | Ground beef | *Staphylococcus* | -1.57 | 3.E-03 |
|  |  |  | *Carnobacterium* | -1.95 | 2.E-03 |
|  |  |  | *Kocuria* | -2.45 | 2.E-06 |
|  |  |  | *Arthrobacter* | -2.68 | 1.E-03 |
|  |  |  | *Pseudomonas* | -4.70 | 2.E-23 |
|  |  |  | *Pantoea* | -5.55 | 1.E-13 |
|  |  |  | *Corynebacterium* | -8.14 | 2.E-39 |
|  |  |  | *Serratia* | -9.05 | 1.E-50 |
|  | July | Non-ground beef | *Methylobacterium* | 21.53 | 1.E-14 |
|  |  |  | *Anoxybacillus* | 20.35 | 1.E-10 |
|  |  |  | *Pseudomonas* | 4.42 | 7.E-03 |
|  |  |  | *Brochothrix* | 3.17 | 5.E-03 |
|  |  |  | *Staphylococcus* | 3.07 | 4.E-03 |
|  |  | Ground beef | *Enterococcus* | -3.78 | 3.E-04 |
|  |  |  | *Corynebacterium* | -8.13 | 5.E-09 |
| **Location C** | January | Non-ground beef | *Pelomonas* | 4.53 | 2.E-04 |
|  |  |  | *Salmonella* | 3.92 | 2.E-04 |
|  |  |  | *Propionibacterium* | 2.41 | 5.E-03 |
|  |  |  | *Deinococcus* | 2.03 | 2.E-04 |
|  |  |  | *Micrococcus* | 1.93 | 8.E-03 |
|  |  | Ground beef | *Kocuria* | -4.11 | 2.E-04 |
|  |  |  | *Brochothrix* | -4.76 | 2.E-04 |
|  | July |  | *N.D* | | |
|  |  |  |  |  |  |

*N.D: Not detected.

| **Supplementary Table S3.** The list of core genus (> 0.01% relative abundance) | | | |
| --- | --- | --- | --- |
|  | **Genus** | **Phylum** | **Mean abundance** ± **SD (%)** |
| **Core genera in all samples** | *Acidovorax* | *Proteobacteria* | 0.09 ± 0.23 |
|  | *Acinetobacter* | *Proteobacteria* | 1.53 ± 1.57 |
|  | *Arthrobacter* | *Actinobacteria* | 0.30 ± 0.72 |
|  | *Atopostipes* | *Firmicutes* | 0.06 ± 0.07 |
|  | *Bacillus* | *Firmicutes* | 0.46 ± 0.58 |
|  | *Bradyrhizobium* | *Proteobacteria* | 0.65 ± 1.43 |
|  | *Brevibacterium* | *Actinobacteria* | 0.04 ± 0.06 |
|  | *Brevundimonas* | *Proteobacteria* | 0.23 ± 0.45 |
|  | *Brochothrix* | *Firmicutes* | 5.17 ± 8.10 |
|  | *Carnobacterium* | *Firmicutes* | 20.37 ± 13.33 |
|  | *Chryseobacterium* | *Bacteroidetes* | 0.09 ± 0.14 |
|  | *Citrobacter* | *Proteobacteria* | 0.10 ± 0.12 |
|  | *Clostridium* | *Firmicutes* | 0.66 ± 1.22 |
|  | *Deinococcus* | *Deinococcus-Thermus* | 0.94 ± 2.16 |
|  | *Delftia* | *Proteobacteria* | 0.01 ± 0.01 |
|  | *Enterobacter* | *Proteobacteria* | 0.18 ± 0.20 |
|  | *Enterococcus* | *Firmicutes* | 0.79 ± 1.27 |
|  | *Erwinia* | *Proteobacteria* | 0.03 ± 0.06 |
|  | *Escherichia* | *Proteobacteria* | 3.36 ± 4.51 |
|  | *Janibacter* | *Actinobacteria* | 0.02 ± 0.03 |
|  | *Janthinobacterium* | *Proteobacteria* | 0.15 ± 0.21 |
|  | *Jeotgalicoccus* | *Firmicutes* | 0.07 ± 0.10 |
|  | *Kocuria* | *Actinobacteria* | 5.10 ± 8.00 |
|  | *Kosakonia* | *Proteobacteria* | 0.07 ± 0.16 |
|  | *Kurthia* | *Firmicutes* | 0.16 ± 0.25 |
|  | *Lactobacillus* | *Firmicutes* | 10.85 ± 12.29 |
|  | *Lactococcus* | *Firmicutes* | 2.03 ± 2.16 |
|  | *Leuconostoc* | *Firmicutes* | 2.11 ± 3.02 |
|  | *Methylobacterium* | *Proteobacteria* | 0.26 ± 0.42 |
|  | *Micrococcus* | *Actinobacteria* | 1.07 ± 2.36 |
|  | *Moraxella* | *Proteobacteria* | 1.16 ± 1.62 |
|  | *Myroides* | *Bacteroidetes* | 0.39 ± 0.77 |
|  | *Ochrobactrum* | *Proteobacteria* | 0.14 ± 0.28 |
|  | *Pantoea* | *Proteobacteria* | 0.53 ± 1.40 |
|  | *Pelomonas* | *Proteobacteria* | 0.54 ± 1.52 |
|  | *Phreatobacter* | *Proteobacteria* | 0.04 ± 0.10 |
|  | *Propionibacterium* | *Actinobacteria* | 1.12 ± 1.45 |
|  | *Proteus* | *Proteobacteria* | 0.07 ± 0.12 |
|  | *Pseudocitrobacter* | *Proteobacteria* | 0.01 ± 0.01 |
|  | *Pseudomonas* | *Proteobacteria* | 13.93 ± 10.51 |
|  | *Psychrobacter* | *Proteobacteria* | 0.59 ± 1.01 |
|  | *Rahnella* | *Proteobacteria* | 1.31 ± 2.21 |
|  | *Rhodococcus* | *Actinobacteria* | 0.02 ± 0.03 |
|  | *Rothia* | *Actinobacteria* | 0.02 ± 0.03 |
|  | *Rouxiella* | *Proteobacteria* | 0.29 ± 0.53 |
|  | *Salmonella* | *Proteobacteria* | 1.48 ± 4.18 |
|  | *Serratia* | *Proteobacteria* | 5.65 ± 8.92 |
|  | *Sphingomonas* | *Proteobacteria* | 0.65 ± 1.40 |
|  | *Staphylococcus* | *Firmicutes* | 1.98 ± 2.85 |
|  | *Streptococcus* | *Firmicutes* | 0.03 ± 0.05 |
|  | *Vagococcus* | *Firmicutes* | 0.08 ± 0.07 |
|  | *Yersinia* | *Proteobacteria* | 0.09 ± 0.14 |
| **Core genera in January samples** | *Aerococcus* | *Firmicutes* | 0.01 ± 0.01 |
|  | *Aeromonas* | *Proteobacteria* | 0.11 ± 0.14 |
|  | *Anoxybacillus* | *Firmicutes* | 1.02 ± 1.66 |
|  | *Catonella* | *Firmicutes* | 0.01 ± 0.01 |
|  | *Caulobacter* | *Proteobacteria* | 0.03 ± 0.05 |
|  | *Corynebacterium* | *Actinobacteria* | 1.67 ± 4.51 |
|  | *CP009312_g* | *Actinobacteria* | 0.03 ± 0.05 |
|  | *Cupriavidus* | *Proteobacteria* | 0.17 ± 0.45 |
|  | *Fulvimonas* | *Proteobacteria* | 0.06 ± 0.18 |
|  | *Glutamicibacter* | *Actinobacteria* | 0.03 ± 0.04 |
|  | *Haemophilus* | *Proteobacteria* | 0.02 ± 0.04 |
|  | *Hathewaya* | *Firmicutes* | 0.02 ± 0.03 |
|  | *Klebsiella* | *Proteobacteria* | 0.01 ± 0.01 |
|  | *Leucobacter* | *Actinobacteria* | 0.01 ± 0.01 |
|  | *Mycoplasma_g14* | *Tenericutes* | 0.04 ± 0.10 |
|  | *Paeniglutamicibacter* | *Actinobacteria* | 0.37 ± 1.05 |
|  | *Paracoccus* | *Proteobacteria* | 0.08 ± 0.14 |
|  | *Peptoniphilus* | *Firmicutes* | 0.90 ± 1.70 |
|  | *Prevotella* | *Bacteroidetes* | 0.01 ± 0.02 |
|  | *Providencia* | *Proteobacteria* | 0.02 ± 0.03 |
|  | *Ralstonia* | *Proteobacteria* | 0.03 ± 0.04 |
|  | *Shewanella* | *Proteobacteria* | 0.05 ± 0.06 |
|  | *Sphingobacterium* | *Bacteroidetes* | 0.01 ± 0.01 |
|  | *Sphingobium* | *Proteobacteria* | 0.12 ± 0.31 |
|  | *Stenotrophomonas* | *Proteobacteria* | 0.04 ± 0.05 |
|  | *Turicibacter* | *Firmicutes* | 0.01 ± 0.01 |
|  | *Vibrio* | *Proteobacteria* | 0.44 ± 0.89 |
| **Core genera in July samples** | *Afipia* | *Proteobacteria* | 0.13 ± 0.34 |
|  | *AJ279038_g* | *Firmicutes* | 0.01 ± 0.02 |
|  | *Aquabacterium* | *Proteobacteria* | 0.01 ± 0.01 |
|  | *Asaccharospora* | *Firmicutes* | 0.01 ± 0.02 |
|  | *Buttiauxella* | *Proteobacteria* | 0.15 ± 0.19 |
|  | *Comamonas* | *Proteobacteria* | 0.06 ± 0.07 |
|  | *Cronobacter* | *Proteobacteria* | 0.01 ± 0.01 |
|  | *Epilithonimonas* | *Bacteroidetes* | 0.02 ± 0.05 |
|  | *Ewingella* | *Proteobacteria* | 0.03 ± 0.05 |
|  | *Exiguobacterium* | *Firmicutes* | 0.02 ± 0.03 |
|  | *Flavobacterium* | *Bacteroidetes* | 0.03 ± 0.05 |
|  | *Hafnia* | *Proteobacteria* | 0.17 ± 0.44 |
|  | *Herbaspirillum* | *Proteobacteria* | 0.09 ± 0.26 |
|  | *JPOM_g* | *Proteobacteria* | 0.69 ± 1.88 |
|  | *Kluyvera* | *Proteobacteria* | 0.02 ± 0.04 |
|  | *Leclercia* | *Proteobacteria* | 0.01 ± 0.03 |
|  | *Lelliottia* | *Proteobacteria* | 0.01 ± 0.03 |
|  | *Macrococcus* | *Firmicutes* | 3.12 ± 4.01 |
|  | *Novosphingobium* | *Proteobacteria* | 0.01 ± 0.02 |
|  | *Pectobacterium* | *Proteobacteria* | 0.03 ± 0.04 |
|  | *Raoultella* | *Proteobacteria* | 0.03 ± 0.05 |
|  | *Trichococcus* | *Firmicutes* | 0.01 ± 0.02 |
|  | *Weissella* | *Firmicutes* | 0.30 ± 0.83 |
|  | *Xanthomonas* | *Proteobacteria* | 0.01 ± 0.02 |
|  | *Yokenella* | *Proteobacteria* | 0.02 ± 0.05 |
| **Core genera in site B samples** | *Aureimonas* | *Proteobacteria* | 0.01 ± 0.02 |
|  | *Planctomicrobium* | *Planctomycetes* | 0.00 ± 0.01 |
| **Core genera in site C samples** | *Pseudoduganella* | *Proteobacteria* | 0.00 ± 0.01 |

| **Supplementary Table 4.** The list of predicted pathways which were significantly different between groups | |  |
| --- | --- | --- |
| **Observation Ids** | **Description** | **Difference between mean proportions (%)** |
| **Group 1** |  |  |
| P105-PWY | TCA cycle IV (2-oxoglutarate decarboxylase) | -0.28 |
| P108-PWY | pyruvate fermentation to propanoate I | -0.21 |
| P23-PWY | reductive TCA cycle I | -0.24 |
| PWY-3781 | aerobic respiration I (cytochrome c) | -0.56 |
| PWY-6969 | TCA cycle V (2-oxoglutarate:ferredoxin oxidoreductase) | -0.29 |
| PWY-7431 | aromatic biogenic amine degradation (bacteria) | -0.21 |
| **Group 2, Group 3, Group 4** |  |  |
| ANAEROFRUCAT-PWY | homolactic fermentation | 0.23 |
| ANAGLYCOLYSIS-PWY | glycolysis III (from glucose) | 0.21 |
| COA-PWY | coenzyme A biosynthesis I | 0.21 |
| GLYCOLYSIS | glycolysis I (from glucose 6-phosphate) | 0.20 |
| P161-PWY | acetylene degradation | 0.33 |
| P562-PWY | myo-inositol degradation I | 0.30 |
| PWY-2941 | L-lysine biosynthesis II | 0.26 |
| PWY-5100 | pyruvate fermentation to acetate and lactate II | 0.29 |
| PWY-5484 | glycolysis II (from fructose 6-phosphate) | 0.21 |
| PWY-5910 | superpathway of geranylgeranyldiphosphate biosynthesis I (via mevalonate) | 0.36 |
| PWY-5973 | cis-vaccenate biosynthesis | 0.23 |
| PWY-6121 | 5-aminoimidazole ribonucleotide biosynthesis I | 0.20 |
| PWY-621 | sucrose degradation III (sucrose invertase) | 0.26 |
| PWY-6609 | adenine and adenosine salvage III | 0.36 |
| PWY-7208 | superpathway of pyrimidine nucleobases salvage | 0.23 |
| PWY-7219 | adenosine ribonucleotides de novo biosynthesis | 0.29 |
| PWY-7229 | superpathway of adenosine nucleotides de novo biosynthesis I | 0.22 |
| PWY-7237 | myo-, chiro- and scillo-inositol degradation | 0.51 |
| PWY-7663 | gondoate biosynthesis (anaerobic) | 0.23 |
| PWY-922 | mevalonate pathway I | 0.35 |
| PWY0-1296 | purine ribonucleosides degradation | 0.26 |
| PWY0-1297 | superpathway of purine deoxyribonucleosides degradation | 0.27 |
| PWY0-1298 | superpathway of pyrimidine deoxyribonucleosides degradation | 0.31 |
| PWY0-1586 | peptidoglycan maturation (meso-diaminopimelate containing) | 0.26 |


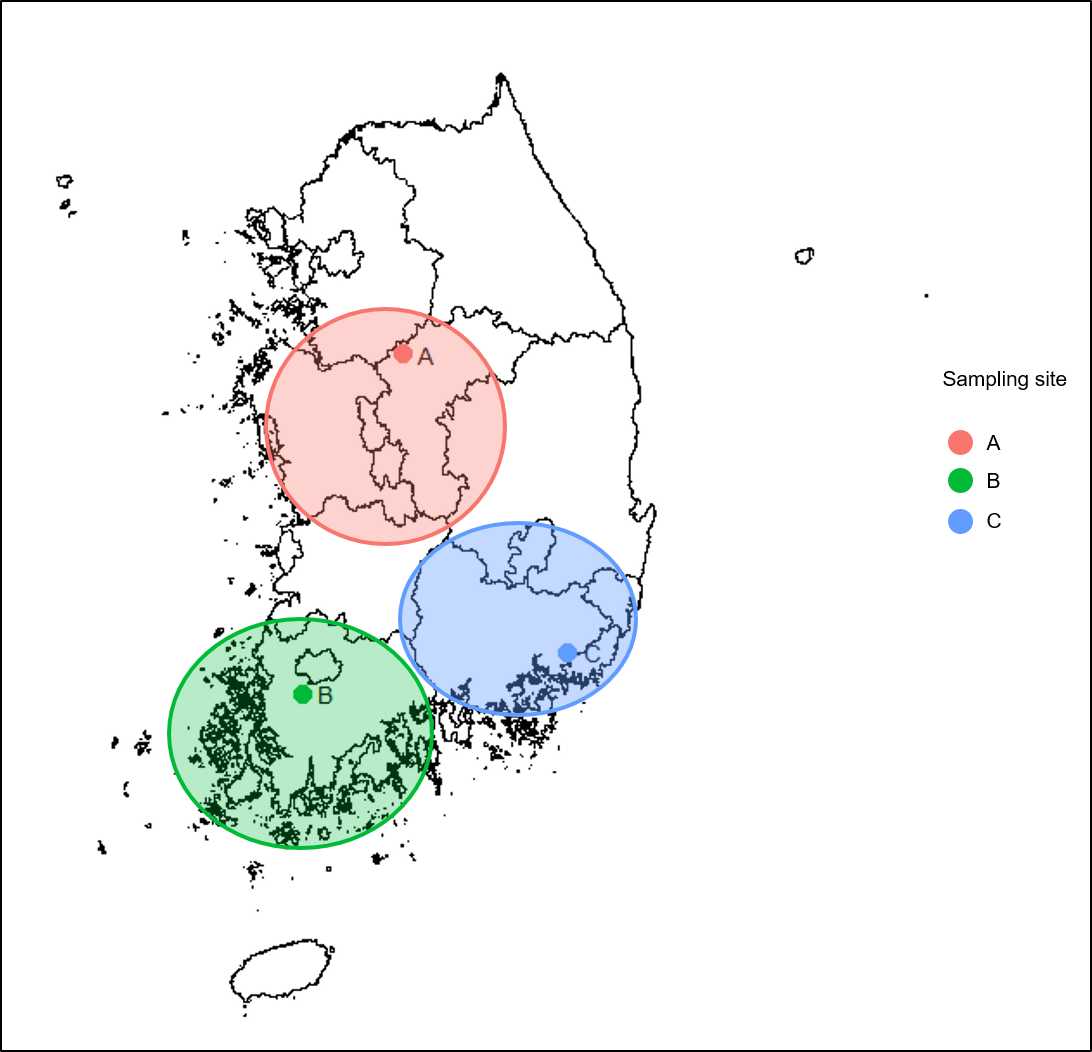


**Supplementary Figure S1.** Sampling sites of beef meat. Samples were obtained from three different sites in January 2018 and July 2018. Those sites are known as the maximum production areas in South Korea. The beef cattle from the local farms in larger circles were transported into each district LPC for processing and transportation to customer.


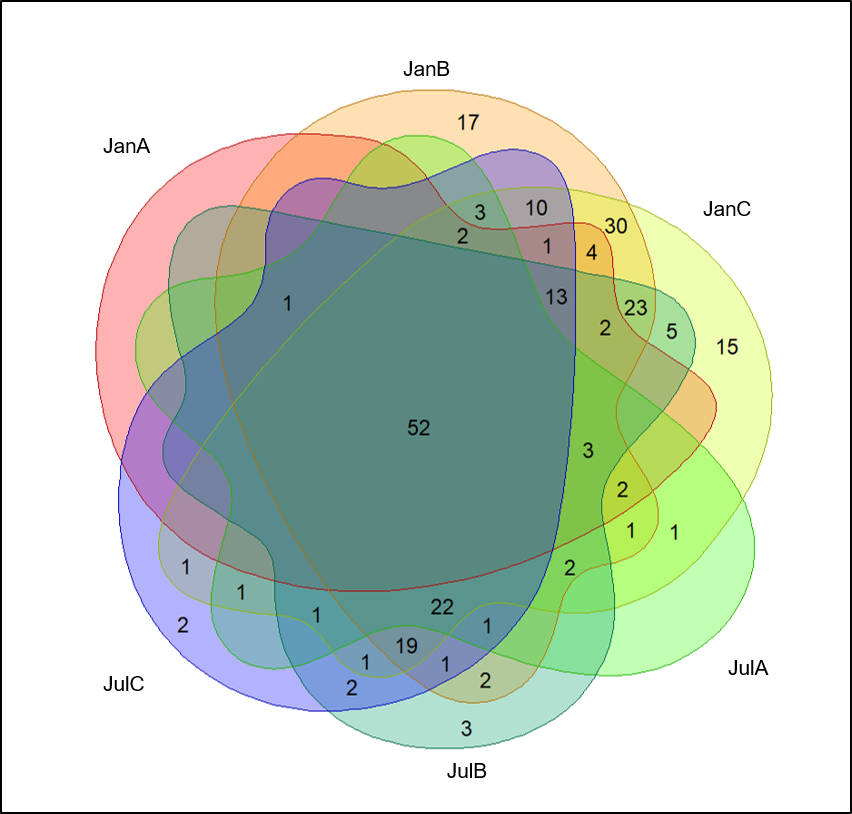
**Supplementary Figure S2**. Core genera of beef microbiota. Venn diagram shows the shared genera among samples in each season. Genera over 0.1 % in the microbiota of each sample were used. Samples were grouped by site in each sampling month. There were 27 core genera in January samples, and 25 core genera in July samples. And 52 genera would be core of beef microbiota. Site-specific genera were found in location B and C with small proportions.
